# Artificial intelligence-based automated matching of pulmonary nodules on follow-up chest CT

**DOI:** 10.1186/s41747-025-00579-w

**Published:** 2025-05-02

**Authors:** Nicola Fink, Jonathan I. Sperl, Johannes Rueckel, Theresa Stüber, Sophia S. Goller, Jan Rudolph, Felix Escher, Theresia Aschauer, Boj F. Hoppe, Jens Ricke, Bastian O. Sabel

**Affiliations:** 1 Department of Radiology, University Hospital, LMU Munich, Munich, Germany; 2 Comprehensive Pneumology Center (CPC-M), Member of the German Center for Lung Research (DZL), Munich, Germany; 3 Siemens Healthineers, Erlangen, Germany; 4 Institute of Neuroradiology, University Hospital, LMU Munich, Munich, Germany; 5 Department of Statistics, Statistical Learning & Data Science, LMU Munich, Munich, Germany; 6 Department of Radiology, Asklepios Lung Clinic Munich-Gauting, Gauting, Germany

**Keywords:** Artificial intelligence, Diagnostic techniques and procedures, Lung neoplasms, Multiple pulmonary nodules, Tomography (x-ray computed)

## Abstract

**Background:**

The growing demand for follow-up imaging highlights the need for tools supporting the assessment of pulmonary nodules over time. We evaluated the performance of an artificial intelligence (AI)-based system for automated nodule matching.

**Methods:**

In this single-center study, patients with nodules and ≤ 2 chest computed tomography (CT) examinations were retrospectively selected. An AI-based algorithm was used for automated nodule detection and matching. The matching rate and the causes for incorrect matching were evaluated for the ten largest lesions (5–30 mm in diameter) registered on baseline CT. The dependence of the matching rate on nodule number and localization was also analyzed.

**Results:**

One hundred patients (46 females), with a median age of 62 years (interquartile range 57–69), and 253 CTs were included. Focusing on the ten largest lesions, 1,141 lesions were identified, of which 36 (3.2%) were other structures incorrectly identified as nodules (false-positives). Of the 1,105 identified nodules, 964 (87.2%) were correctly detected and matched. The matching rate for nodules registered in both baseline and follow-up scans was 97.8%. The matching rate per case ranged 80.0–100.0% (median 90.0%). Correct matching rate decreased in follow-up examinations to over 50 nodules (*p* = 0.003), with an overrepresentation of missed matching. Matching rates were higher in parenchymal (91.8%), peripheral (84.4%), and juxtavascular (82.4%) nodules than in juxtaphrenic nodules (71.1%) (*p* < 0.001). Missed matching was overrepresented in juxtavascular, and incorrect assignment in juxtaphrenic nodules.

**Conclusion:**

The correct automated-matching rate of metastatic pulmonary nodules in follow-up examinations was high, but it depends on localization and a number of nodules.

**Relevance statement:**

The algorithm enables precise follow-up matching of pulmonary nodules, potentially providing a solid basis for standardized and accurate evaluations. Understanding the algorithm’s strengths and weaknesses based on nodule localization and number enhances the interpretation of AI-based results.

**Key Points:**

- The AI algorithm achieved a correct nodule matching rate of 87.2% and up to 97.8% when considering nodules detected in both baseline and follow-up scans.
- Matching accuracy depended on nodule number and localization.
- This algorithm has the potential to support response evaluation criteria in solid tumor-based evaluations in clinical practice.

**Graphical Abstract:**

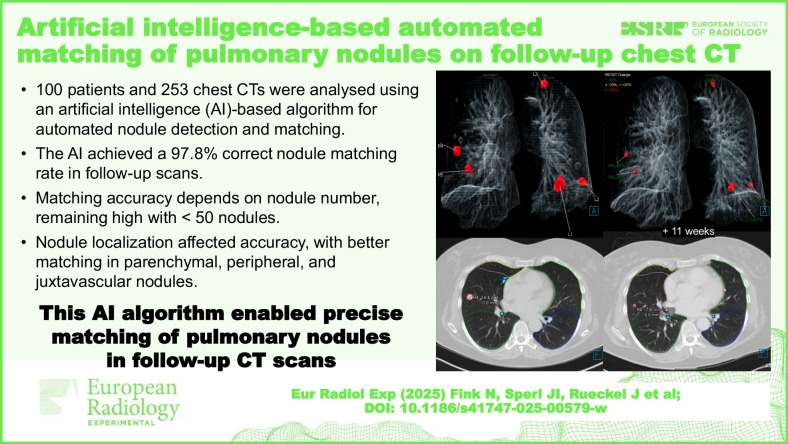

## Background

Computed tomography (CT) is considered the standard of care for detecting pulmonary nodules [1]. In recent years, CT technology has undergone several improvements, including higher image resolution and low-dose acquisition, resulting in a more sensitive detection of pulmonary nodules and a higher number of scans being performed [2, 3]. Thus, radiologists are increasingly confronted with incidental findings, including pulmonary nodules that may require follow-up to further determine their nature [4–6]. Although approximately 95% of those nodules are benign [5, 7], it is crucial to assess all of them appropriately, if necessary with follow-up CT scans [4], in order to detect the malignant ones at an early stage.

Furthermore, the incidence of lung cancer continues to rise, further driving the need for follow-up CT examinations to monitor pulmonary nodules. This gets even more relevant in nodules detected as part of CT-based lung cancer screening performed in high-risk individuals. Several randomized trials have demonstrated the added value of lung cancer screening using low-dose CT by reducing lung cancer mortality significantly [8–13] and thus support its implementation for a clearly defined risk population. At the same time, this potentially results in a relatively high number of false-positive results [14] and subsequently an increasing number of follow-up examinations that require standardized assessment. A systematic review of, among others, eight randomized lung cancer trials showed that pulmonary nodules were detected in up to 51% of the included patients [15].

Standardized reporting of changes in size, number, and morphologic features plays an important role not only in indeterminate pulmonary nodules [16] but also in assessing treatment response in cancer patients to guide further patient management [17–19]. While computer-aided detection (CAD) systems primarily increase nodule detection sensitivity and already have been shown to reduce the individual reading time [20–22], matching and comparing a pulmonary nodule between two successive examinations is particularly time-consuming and tedious. Ideally, this should be conducted according to standardized reporting systems, such as the lung imaging reporting and data system (Lung-RADS) [23] and the response evaluation criteria in solid tumors (RECIST) [24]. In this context, it has already been demonstrated that the involvement of a specialized technologist, performing the subsequent measurement during follow-up instead of the radiologist, reduces radiologists’ reading time by 87% [25]. Using an artificial intelligence (AI) algorithm instead of an additional human would make this task even more cost-effective, as AI-based automated matching also improves diagnostic efficiency [26].

Hence, the fully automatic identification of the same nodule(s) over multiple time points, hereinafter referred to as nodule matching, has the potential to further improve the follow-up of pulmonary nodules, and thus may increase radiologists’ efficiency and accuracy when interpreting serial chest CT scans.

This study aimed to evaluate the performance of an AI-based system performing automated matching of pulmonary nodules in successive CT scans of the chest and to assess its dependence on nodule localization and number.

## Methods

The protocol of this retrospective, single-center study was approved by the local institutional Review Board, which waived requirements for informed consent (see “Declarations”).

### Patient population

Data from patients with an underlying malignant disease, at least one pulmonary nodule, and two or three successive chest CT scans were retrospectively and randomly selected. Patient selection was performed by screening our Picture Archiving and Communication System (PACS) using keywords indicative of pulmonary nodules. The cases were manually reviewed to confirm the presence of pulmonary nodules and to ensure that all inclusion criteria were met. Cases with exclusion criteria were removed from this cohort. Baseline characteristics of the study cohort were derived from medical records, including patient demographics and the underlying malignant disease. The exclusion criteria were as follows: (i) age < 18 years and (ii) thoracic surgery in between the included CT scans. Due to the inclusion criteria, included nodules were primarily metastatic.

### Image acquisition and reconstruction

CT scans were performed on Somatom Force, Somatom Definition AS+, Somatom Definition Flash, Somatom Drive (all Siemens Healthineers, Forchheim, Germany), or Optima 660 (GE Healthcare, Chicago, IL, USA) scanners. Patients underwent either an unenhanced high-resolution or a contrast-enhanced CT scan using a standardized institutional protocol. Key parameters included a tube voltage of 100–120 kVp, tube current modulation (100–300 mAs), rotation time of 0.5–0.6 s, and pitch of 0.9–1.2. Reconstructed in-plane resolution for axial images ranged from 0.5 × 0.5 mm to 0.7 × 0.7 mm, depending on the scanner and patient size. Images were reconstructed using iterative reconstruction techniques, including advanced modeled iterative reconstruction—ADMIRE) on Siemens Healthineers scanners and Adaptive Statistical Iterative Reconstruction—ASiR on the GE Optima 660 scanner. Standard soft tissue kernels were used for general assessment, while high-spatial-resolution lung kernels were applied for detailed nodule evaluation. Image analysis was conducted on a RadiForce RX370 monitor (EIZO, Hakusan, Ishikawa, Japan) (3 megapixels, Digital Imaging and Communications in Medicine (DICOM)-calibrated, maximum brightness 1,000 cd/m², contrast ratio 1,000:1) under electronically controlled optimal lighting conditions (ambient light < 25 lux).

### Automatic nodule matching

All data sets were analyzed using the cloud-based prototype “AI-Rad Companion Research Chest CT Explore” (Siemens Healthineers), involving four subsequent steps:

1. AI-based detection of lung nodules (LungCAD, VD20);
2. AI-based extraction of anatomical landmarks, including a mesh of the lung lobes [27–29];
3. affine co-registration of the landmarks over multiple time points using an iterative closest point algorithm [30]; and
4. identification of nodule pairs in the coregistered space.

**Fig. 1:**
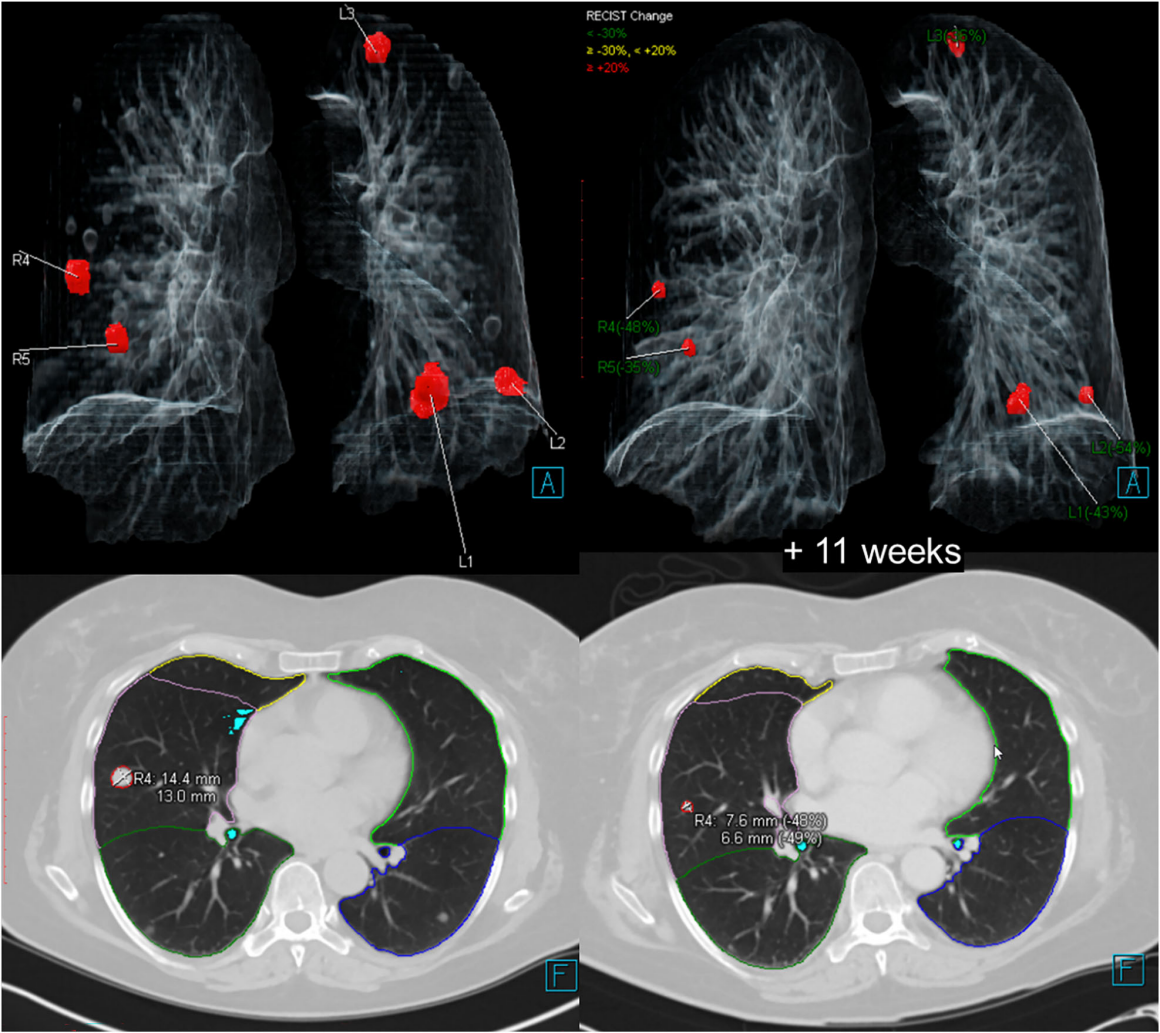
Sample case of the visualization of the AI-based results for automated matching

The third step is a data-minimalistic approach suitable for cloud-based processing where the full three-dimensional data set of the prior study is no longer available in the cloud at the time point of the follow-up exam. In the fourth step, the Euclidean distance is calculated for the coregistered nodules, and a pair is considered a match if the distance is below 15 mm. This distance threshold was established by analyzing the matching performance on an independent cohort. Figure 1 illustrates a sample case for the visualization of the AI-based results. This algorithm was a prototype at the time of initial evaluation but is now commercially available as part of the AI-Rad Companion Chest CT product (version VA20, Siemens Healthineers). The nodule detection component has been shown to have high sensitivity and specificity in prior studies [31–33].

Overall, the underlying AI is the anatomical landmark detection, as well as lung lobe segmentation. Anatomical landmark detection has been trained on over 15,000 CT data sets [27]. The lung lobe segmentation U-Net has been trained on over 5,000 CT data sets [31].

### Radiological reading and algorithm performance

Regardless of whether the included patients received two or three CT scans, each follow-up was evaluated separately, resulting in 153 follow-up examinations. The entire process of nodule detection and segmentation was fully automated. The validation of the AI algorithm’s results was performed by a radiologist with an experience of five years in thoracic imaging (N.F.), whose role was to identify false positives, to verify whether the nodules matched by the AI corresponded to the same nodules on follow-up scans and to identify the root causes of incorrect matching. Nodules missed during detection were not included in the analysis, as the primary aim of this study was to assess the algorithm’s matching performance. The radiologist received the anonymized CT data as axial reconstructions, as well as AI results (Fig. 1), and was allowed to perform various analyses, utilizing standard tools available in a conventional DICOM viewer, such as size measurement, region-of-interest placement, and density analysis. The analysis was focused on a maximum of ten pulmonary nodules (size between 5 mm and 30 mm in diameter) found by the AI algorithm in the baseline CT scan. Nodules that had completely disappeared on follow-up CT and subsolid nodules, including mixed (solid and subsolid) nodules, were excluded. For each lesion assigned as “incorrectly detected/matched” the root cause was documented as follows:

1. false-positive detection (other, non-nodular structures, such as atelectasis, vertebral bodies, or ribs, incorrectly identified as pulmonary nodules by the algorithm)
2. missed matching (nodule registered at baseline but not in follow-up CT despite its presence); and
3. incorrect assignment (nodule present in both baseline and follow-up scans but incorrectly linked to a different nodule instead of its true counterpart).

In addition, the total number of pulmonary nodules was documented, and the selected pulmonary nodules were classified according to their localization as parenchymal, peripheral, juxtavascular, or juxtaphrenic. Peripheral nodules were defined as being located within 10 mm of the pleura, juxtavascular and juxtaphrenic nodules as directly adjacent to a vessel and the diaphragm, respectively.

### Statistical analysis

The software GraphPad Prism (version 8.4.2, GraphPad, San Diego, CA, USA) and R-Studio (version 1.4.1717, RStudio Inc., Boston, MA, USA) were used for statistical analyses and graphical illustration. Continuous variables are reported as median with interquartile range, categorical variables as absolute frequencies and relative frequencies.

Based on a binary outcome (correct or incorrect), we evaluated the matching rate depending on nodule number and localization. The association between nodule number and matching rate, as well as between nodule localization and matching rate, each including root causes for incorrect matching, has been screened for independence by χ^2^ analysis based on contingency tables including Pearson residuals. Mann–Whitney *U*-test was used to evaluate differences in matching rates between follow-ups with different numbers of pulmonary nodules.

Any test result with a *p*-value smaller than 0.05 was considered statistically significant.

## Results

### Patient population

The study population included 100 patients (46 females) with a median age of 62 years (interquartile range 57–69), with an underlying malignant disease and pulmonary nodules. The most frequent origin of the underlying malignant disease was colorectal (*n* = 24), followed by thyroid carcinoma (*n* = 17), sarcoma (*n* = 12%), and malignant melanoma (*n* = 11). Six patients had signs of lung emphysema, but none of the included patients had lung fibrosis. A total of 253 thoracic CT scans were included (Table 1). With some patients having two and some three CT scans, this resulted in a total of 153 follow-up examinations. The median time between scans per follow-up was 13.7 (range 11.1–18.5) weeks. Detailed baseline characteristics of all included patients are shown in Table 2.

**Table 1:** Type of CT examinations and radiation doses

	Total scans (*n* = 253)
Use of intravenous contrast agent	
Unenhanced	51 (20.2)
Contrast-enhanced	202 (79.8)
Initial examination range	
Neck and thorax	3 (1.2)
Neck, thorax, and abdomen	36 (14.2)
Thorax and abdomen	129 (51.0)
Thorax	85 (33.6)
Radiation dose	
CTDI (mGy)	6.7 (5.1–8.5)
DLP	387.0 (267.9–527.6)

Values are median with interquartile range or *n* (%)

*CTDI* Computed tomography dose index, *DLP* Dose length product

**Table 2:** Patient and pulmonary lesion characteristics

Clinical characteristics		Total (*n* = 100)
Age in years, median (IQR)		62.0 (57.0–69.0)
Sex, *n* (%)	Female	46 (46.0)
	Male	54 (54.0)
Underlying malignant disease, *n* (%)	Colorectal cancer Thyroid cancer Sarcoma Malignant melanoma Hepatocellular/cholangiocarcinoma Pancreatic cancer Urologic cancer Breast cancer Pharyngeal cancer Hemangioendothelioma Gynecological cancer Upper gastrointestinal cancer Cancer of unknown primary Parotid cancer Prostate cancer	24 (24.0) 17 (17.0) 12 (12.0) 11 (11.0) 6 (6.0) 6 (6.0) 6 (6.0) 5 (5.0) 3 (3.0) 3 (3.0) 2 (2.0) 2 (2.0) 1 (1.0) 1 (1.0) 1 (1.0)
Pulmonary lesions		Total (*n* = 1,141)
	False-positives	36 (3.2)
	Pulmonary nodules	1,105 (96.8)
	Parenchymal	549 (49.7)
	Peripheral	346 (31.3)
	Juxtavascular	165 (14.9)
	Juxtaphrenic	45 (4.1)

*IQR* Interquartile range

### Automatic nodule matching

Focused on the ten largest lesions registered in baseline CT scan per follow-up examination by the algorithm, a total of 1,141 lesions were included for further analysis. Among these, 36 lesions (3.2%) were false positives, meaning they were other, non-nodular structures (*e.g*., atelectasis, vertebral bodies, ribs) incorrectly identified as pulmonary nodules in the baseline scan. Among the remaining 1,105 pulmonary nodules, 141 (12.8%) were not correctly matched at follow-up, with the following root causes: 119 (10.8%) were missed at follow-up (detected at the baseline but not in the follow-up scan despite being present; “missed matching”) and 22 (2.0%) were incorrectly assigned (two different nodules were erroneously matched; “incorrect assignment”). This resulted in 964 nodules that were both correctly identified at baseline and correctly matched at follow-up, leading to a correct nodule matching rate of 87.2% (964/1105). The median correct matching rate per follow-up was 90.0% (80.0–100.0%).

Additionally, considering only nodules that were detected as pulmonary nodules in both CT scans (baseline and follow-up), a total of 986 nodules were included. Among these, 964 were correctly matched, resulting in a correct nodule matching rate of 97.8% (964/986).

**Fig. 2:**
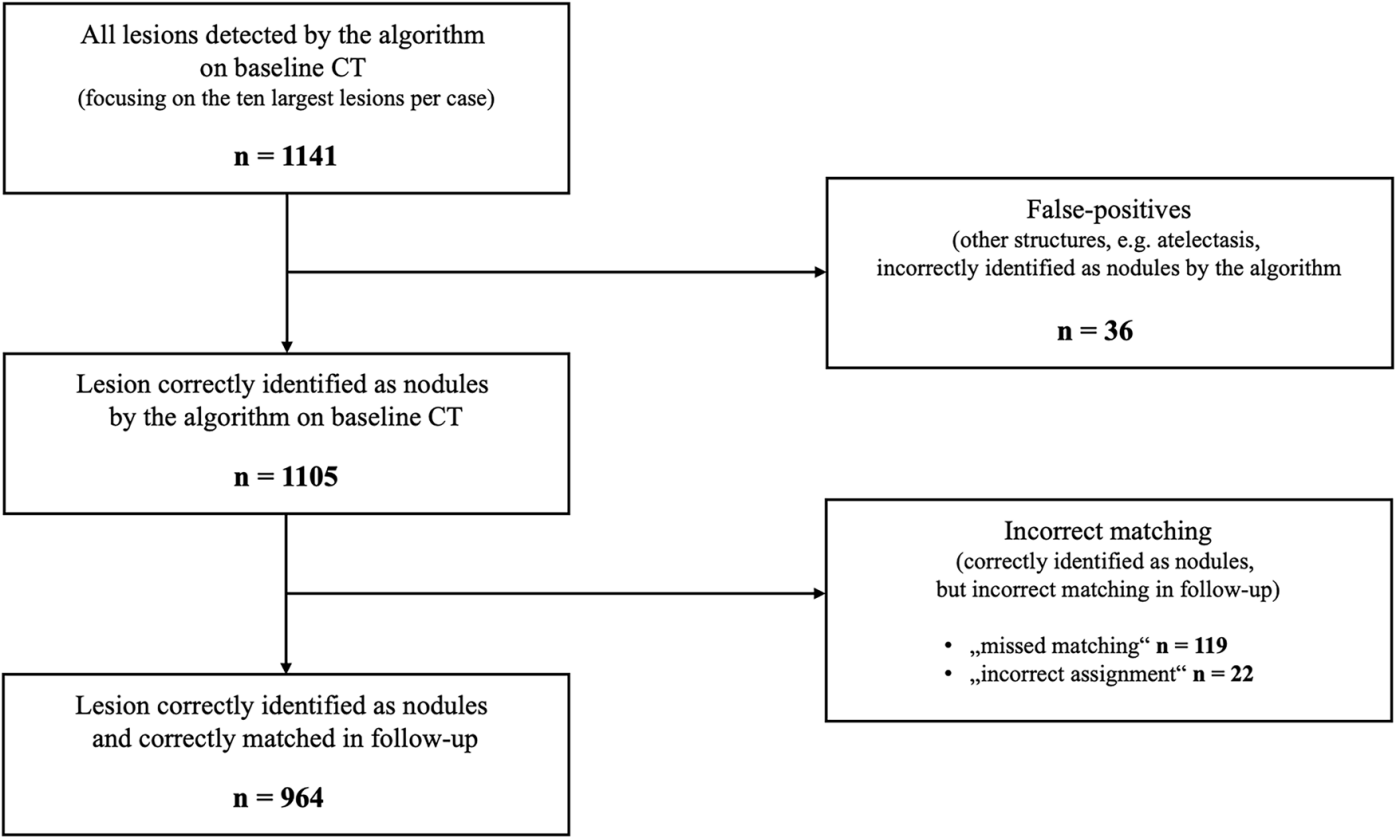
Flowchart illustrating nodule categorization

Figure 2 shows a flowchart that illustrates nodule categorization. The median size of included pulmonary nodules was 9.0 [IQR 7.1–11.7] mm.

### Performance depending on the number and localization of pulmonary nodules

**Fig. 3:**
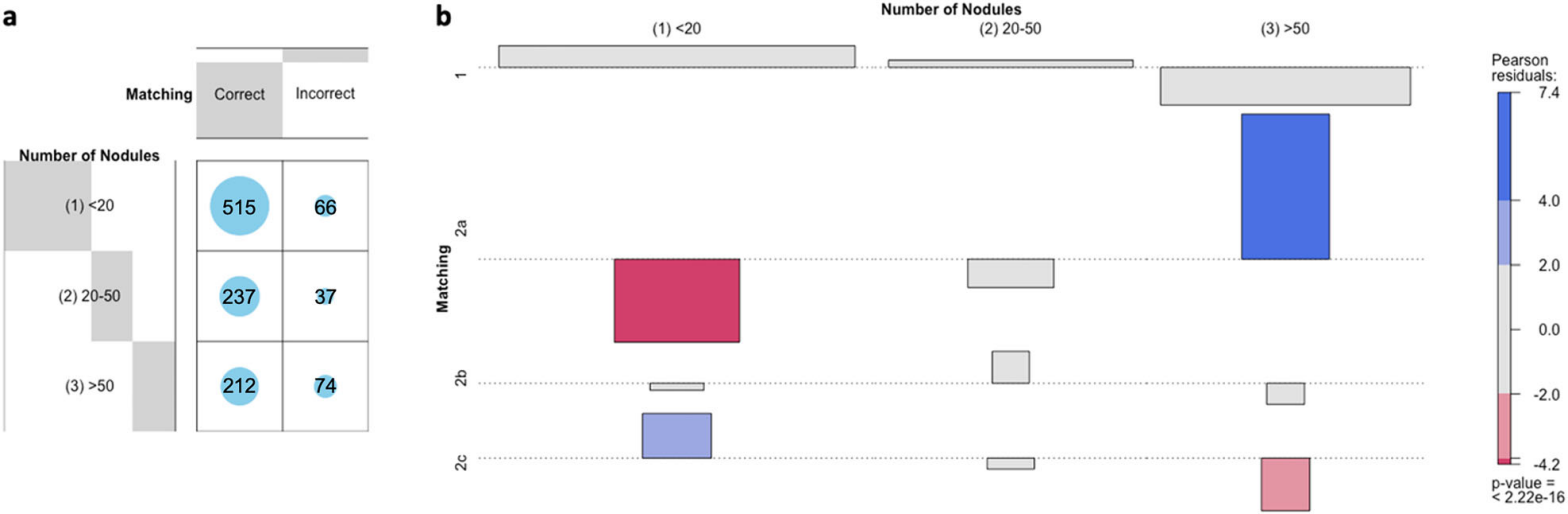
Matching depending on the number of nodules: **a** distribution of the matching (correct *versus* incorrect) per number of pulmonary nodules illustrated as a balloon plot. **b** Cohen-friendly association plot for the relation between nodule number per follow-up and matching rate (1 = correct; 2 = incorrect), as well as root causes for incorrect matching (2a = missed matching; 2b = incorrect assignment; 2c = false-positive detection) illustrating the deviation from the statistically expected contingency table (over-/underrepresentation in red/blue; independency testing using χ^2^ analysis)

Among the 153 included follow-up examinations, 93 (60.8%) had less than 20 and 31 (20.3%) had 20 to 50, and 29 (19.0%) had more than 50 pulmonary nodules. The correct matching rate differed depending on the number of pulmonary nodules (*p* = 0.003). The matching rate was similar in follow-ups with less than 20 nodules (median 100.0%, range 80.0–100.0%) and 20–50 nodules (90.0%, 80.0–100.0%) (*p* = 0.372). It was significantly lower in examinations with more than 50 pulmonary nodules (median 80.0%, 65.0–90.0%) than in those with < 20 nodules (*p* < 0.001) or those with 20–50 nodules (*p* = 0.025). Figure 3 illustrates independence testing of nodule number per follow-up and matching rate, as well as root causes for incorrect matching. Missed matching was significantly overrepresented in follow-ups with more than 50 nodules and significantly underrepresented in follow-ups with less than 20 nodules. At the same time, false-positive detection of non-nodule structures was overrepresented in follow-ups with less than 20 and underrepresented in those with more than 50 nodules.

**Fig. 4:**
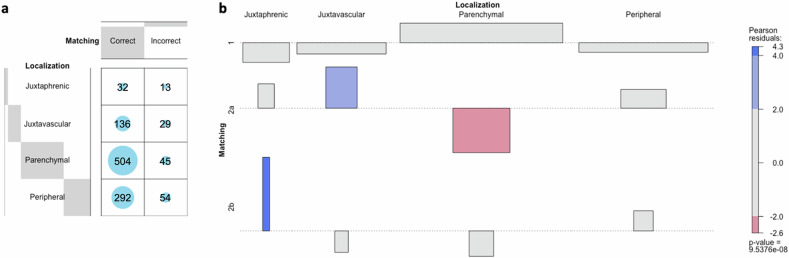
Matching depending on the localization of nodules: **a** distribution of the matching (correct *versus* incorrect) per localization of the pulmonary nodules illustrated as a balloon plot. **b** Cohen-friendly association plot for the relation between nodule localization and matching rate (1 = correct; 2 = incorrect), as well as root causes for incorrect matching (2a = missed matching; 2b = incorrect assignment) illustrating the deviation from the statistically expected contingency table (over-/underrepresentation in red/blue; independency testing using χ^2^ analysis)

Among the pulmonary nodules correctly identified as such on baseline CT (*n* = 1,105), 549 (49.7%) were parenchymal, 346 (31.3%) peripheral, 165 (14.9%) juxtavascular, and 45 (4.1%) juxtaphrenic nodules. The correct matching rate of pulmonary nodules differed significantly depending on the localization (*p* < 0.001). While parenchymal nodules were correctly matched in 91.8% (504/549), the matching rate of peripheral and juxtavascular nodules was 84.4% (292/346) and 82.4% (136/165), respectively. Juxtaphrenic nodules showed the lowest matching rate (71.1%, 32/45). Figure 4 shows independency testing of the nodule localization and the matching rate, as well as the root causes for incorrect matching. While mis-matching was significantly underrepresented in parenchymal nodules, it was overrepresented in juxtavascular nodules. Incorrect assignment of two non-identical nodules was significantly overrepresented in juxtaphrenic nodules.

## Discussion

This study evaluated the performance of the cloud-based prototype AI-Rad Companion Research Chest CT Explore (Siemens Healthineers) for automated matching of pulmonary nodules in serial CT scans. The main findings are as follows: first, this algorithm showed a high correct matching rate; second, the correct matching rate was significantly lower in examinations with more than 50 pulmonary nodules; third, the correct matching rate was significantly higher in parenchymal, peripheral, and juxtavascular than in juxtaphrenic nodules with an overrepresentation of missed matching in juxtavascular and incorrect assignment in juxtaphrenic nodules.

Previous studies evaluated such automated nodule matching in a limited number of patients (from 11 to 40) [34–36], whereas this study investigated matching rates, as well as detailed root causes of decreasing accuracy in a larger cohort of 100 patients. With a median per case matching rate of 90.0%, the algorithm evaluated in the present study showed high performance regarding automated nodule matching in a heterogeneous cohort in terms of localization and the number of pulmonary nodules. Considering only lesions in which the algorithm registered nodules on both CT scans, the nodule matching rate was 97.8%.

This high matching accuracy has important clinical implications. Reliable tracking of nodule changes over time supports treatment decisions by ensuring consistency in monitoring growth or stability and facilitates adherence to standardized protocols such as RECIST and Lung-RADS. Additionally, automated matching reduces the risk of false positives and false negatives, helping to minimize unnecessary interventions and improve diagnostic confidence.

Those matching rates are comparable to or even higher than those reported in previous studies, which were 92.7% [36], 86.3% [34], and 66.7% [35]. Tao et al [36] already attributed their relatively high matching rate compared with other studies to differences in the study population: While their population was based on a lung cancer screening cohort, the other two studies included cancer patients in whom the lung parenchyma may be more damaged due to pre-existing conditions and previous treatments and thus more difficult to assess than in screening patients, most of whom are likely to be relatively healthy. However, in the present study, the correct matching rate did not significantly differ between patients with or without lung parenchymal changes, such as scars and atelectasis. Only a small subset of our study cohort had such conditions, and in cases where they were present, the extent was relatively low. For example, lung fibrosis was not detected in any of the patients. Compared to the results presented in the present study, Lee et al [35] obtained a lower matching rate of 66.7% and only succeeded in raising this to a comparable value of 82.4% when considering only patients with unchanged findings.

The matching rate in the present study was dependent on the number of pulmonary nodules, with a significantly lower matching rate in follow-up examinations with more than 50 nodules. Lee et al [35] already demonstrated decreasing accuracy in automated nodule matching with an increasing number of nodules, mainly caused by unmatched (“missed matching”) rather than incorrectly assigned nodules most likely due to the occupancy of lung parenchyma by a large number of nodules that complicate nodule registration by covering anatomic landmarks. We support this finding with a significant overrepresentation of missed matching in follow-ups with more than 50 nodules, due to the increased complexity associated with a larger number of nodules, possibly resulting in a more challenging anatomical registration during image processing. At the same time, follow-up examinations with less than 20 nodules showed an overrepresentation of false-positives, primarily based on the inclusion of the ten largest lesions at baseline: examinations with a low number of nodules are more likely to contain false-positives among these ten lesions.

Our matching rate was highly associated with the nodule’s localization, with the highest rate of 91.8% in parenchymal nodules, mainly surrounded by low-attenuating lung parenchyma, which provides a strong contrast for both initial detection and subsequent matching. Conversely, juxtavascular nodules exhibited a lower matching rate (due to their proximity to blood vessels), as previously reported in CAD system performance studies [37, 38]. The difficulty in detection may compromise the subsequent registration process. Juxtaphrenic nodules presented the lowest matching rate, primarily due to the effects of diaphragm motion during breathing, explaining the overrepresentation of incorrect assignment. Image-based registration might show superior results in this case, but is not suitable for the cloud-based data-minimalistic approach of the AI-Rad Companion, as the full three-dimensional data set of the baseline exam is not available in the cloud at the time of analysis of the follow-up exam. In contrast to the present study, Beyer et al [34] did not observe differences in matching rates depending on nodule localization. However, they only investigated nodule localization in terms of lung side (right or left lung) and area (upper, middle, or lower third; central or peripheral), not their relation to adjacent structures.

Beyond the high matching accuracy demonstrated in this study, the AI system holds potential for substantial time savings in clinical workflows. Prior research demonstrated that delegating nodule comparison tasks to technologists reduced radiologists’ time by up to 87% [39]. Fully automated AI solutions could achieve similar or greater efficiencies, particularly in high-nodule-count scenarios [25]. Future research should aim to directly quantify these time savings and assess their impact on clinical workflow.

Overall, this automated matching algorithm has the potential to support clinical assessment of incidental and metastatic pulmonary nodules on follow-up examinations. Despite a slight decrease in the correct matching rate in cases with high nodule count or juxtaphrenic nodules, this algorithm works reliably and accurately in most cases. In addition, we included data from multiple CT scanners and imaging protocols, reflecting the variability encountered in routine clinical practice that potentially influences nodule detection and characterization [40, 41]. Despite these challenges, the algorithm demonstrated robust matching performance, which highlights its applicability in heterogeneous clinical environments.

In a real-world scenario, using this algorithm may not only reduce reporting time and improve reporting quality but also provide a basis for standardized evaluations such as Lung-RADS and RECIST-based reporting, in particular in patients undergoing serial imaging for metastatic disease. This approach would reduce the time radiologists spend visually comparing scans and ensure more standardized and reproducible assessments, and minimize variability in follow-up recommendations, enhancing decision-making and facilitating multidisciplinary communication.

The current AI system presented here is designed as a fully automated solution, but could also be adapted for semi-automated workflows that integrate manual input, such as RECIST-based identification of target lesions. By providing a standardized and reliable matching process, the algorithm may help track changes in nodule size more consistently, supporting more accurate assessments of disease progression or treatment response. Future studies should explore how such AI systems can further streamline follow-up evaluations, enhance decision-making accuracy, and ultimately contribute to improved patient outcomes.

This study has several limitations. First, only the ten largest solid nodules per follow-up with a size between 5 mm and 30 mm were included to enable a systematic and reproducible evaluation of the algorithm’s performance. However, since some of the included patients have more than several hundred pulmonary nodules, this may not fully capture the algorithm’s ability to match all detected nodules. Automated matching of very small, very large, or ground-glass nodules may be more complicated and should be assessed in future studies. Second, as this study analyzed the correct matching of pulmonary nodules between two successive CT scans, nodules that disappeared in the follow-up scan were excluded from further analysis. Additionally, while longitudinal tracking across multiple follow-ups is clinically important, only a small subset of our cohort allowed for such analysis, which limited the statistical power for meaningful conclusions. Longitudinal evaluations also involve additional complexities, such as managing new or disappearing nodules and accounting for imaging variability, requiring dedicated analytical frameworks. Third, we did not evaluate the impact of inspiration level or nodule size and growth on the matching rate. The analyzed patient cohort did not include cases with fibrosis, and such parenchymal changes may impact the algorithm’s performance, which should be investigated in future studies. In addition, the distribution of malignancies in this study cohort does not specifically reflect cancer prevalences in the general population, which may affect algorithm performance. Fourth, the validation of the algorithm’s results was conducted by a single experienced radiologist, who focused on cross-checking the AI results rather than independent nodule identification and analysis. Fifth, analyses were only retrospectively performed and based on single-center data. Sixth, the impact of automated nodule matching on clinical workflow and individual patient management was not investigated and should be part of further analyses. Seventh, this study is based on different CT scanners from different CT vendors, as variability between scans often reflects real-world scenarios due to factors such as scanner availability and location. However, it should be emphasized that this study attempts to address the generalizability of our algorithm performance considering scanner variability. Eighth, some patients had multiple follow-up scans; for example, 53 patients had three consecutive CT scans in our study, which may introduce variance and bias in contrast to patients scanned only once or twice, thus potentially affecting the overall algorithm performance.

In conclusion, this study demonstrated that the accuracy of this algorithm (AI-Rad Companion Research Chest CT Explore [Siemens Healthineers]) in automated matching of pulmonary metastatic nodules is high, when focusing on the ten largest nodules per case. However, the algorithm’s performance depends on the localization and number of nodules. Especially the decreasing matching rate in CT scans with high nodule count has to be taken into account when interpreting AI-based results. Overall, the analyzed AI-based solution for automated matching of pulmonary nodules on follow-up chest CT can provide valuable support for standardized reporting in clinical practice.

## Data Availability

The datasets used and/or analyzed during the current study are available from the corresponding author on reasonable request.

## Abbreviations

AI: Artificial intelligence
CAD: Computer-aided detection
CT: Computed tomography
DICOM: Digital imaging and communications in medicine
Lung-RADS: Lung imaging reporting and data system
RECIST: Response evaluation criteria in solid tumors
